# Functional characterization of engineered bacterial biosensors for kynurenine detection

**DOI:** 10.1099/acmi.0.001031.v5

**Published:** 2025-08-08

**Authors:** Pisit Charoenwongwatthana, Halah Ahmed, Wojciech Cajdler, Jamie Coulter, Chien-Yi Chang

**Affiliations:** 1 School of Dental Sciences, Faculty of Medical Sciences Newcastle University, Framlington Place, Newcastle upon Tyne, NE2 4BW, UK; 2 Department of Oral Medicine and Periodontology, Faculty of Dentistry, Mahidol University, Bangkok, Thailand

**Keywords:** biosensor, bioengineering, kynurenine, synthetic biology, tryptophan

## Abstract

The kynurenine (KYN) pathway is the major catabolic pathway for tryptophan in humans, producing several metabolites that influence health. In clinical settings, KYN levels serve as a valuable biomarker for the diagnosis and prognosis of inflammatory and neurological diseases. Nevertheless, KYN detection relies on mass spectrometry analysis, which requires specialized knowledge and expertise with high operational costs. The bacterial biosensor presents as a promising tool for rapid and cost-effective targeted substance detection due to its ease of genetic modification. Therefore, this study aimed to develop an engineered bacterial biosensor by integrating a genetic module in a plasmid designed for KYN detection harboured in an *Escherichia coli* chassis. The KYN biosensing component in the genetic module encodes a KYN pathway regulator (KynR) from *Pseudomonas aeruginosa*, driven by the *P_BAD_* arabinose-inducible promoter. Upon expression, KynR would bind to the exogenous KYN and the bacterial responding *kyn* promoter to express the downstream green fluorescent protein gene to emit a fluorescence signal. However, despite successful induction by arabinose and the presence of KYN, biosensors with different gene orientations and genetic components failed to produce a significant fluorescence signal. These findings suggest that the sensitivity of *P. aeruginosa* KynR is insufficient to detect physiological levels of KYN. Further exploration of alternative biological sensing components is warranted.

## Data Summary

The plasmid designs, bio-component sequences and raw data on bacterial growth and fluorescence production in this study are provided in the supplementary materials. The GenBank accession numbers for KYNvA and KYNvB sequences are PV413183 and PV413184, respectively.

**Impact statement**

This research aimed to develop bacterial biosensors intended for kynurenine (KYN) detection, a potential biomarker for various inflammatory and neurological diseases. Despite optimization of plasmid design and experimental methodology, the biosensors failed to generate a significant fluorescence signal in response to exogenous KYN. These findings highlight that the bacterial KYN pathway regulator may not provide sufficient sensitivity for biosensor construction to detect physiologically relevant KYN concentrations. Further research is required to explore the efficient biosensing elements to develop an applicable biosensor for screening physiological KYN levels in clinical settings.

## Introduction

The kynurenine (KYN) pathway is the major catabolic route for the essential amino acid, tryptophan (Trp) in humans, accounting for ~95% of Trp catabolism [1]. Trp-KYN catabolism is initiated and rate-limited by either tryptophan 2,3-dioxygenase (TDO) (EC 1.13.11.11) or the two isoforms of indoleamine 2,3-dioxygenase (IDO1 and IDO2) (EC 1.13.11.52). KYN is further catabolized through multiple enzymatic reactions, generating several metabolites involved in physiological processes in humans, including kynurenic acid (KYNA), anthranilic acid (AA), 3-hydroxy-l-kynurenine, quinolinic acid (QA) and picolinic acid [2]. QA could be further used for the synthesis of nicotinamide adenine dinucleotide (NAD^+^), which is an essential cofactor involved in various redox reactions [1]. Since the KYN pathway occurs in various cell types, its dysregulation has been implicated in the pathogenesis of several diseases such as neuropsychiatric disorders, neurodegenerative diseases, autoimmune diseases and cancer [2,3]. For instance, high levels of serum KYN have been associated with the poor prognosis of patients with hepatocellular carcinoma [4]. Patients with schizophrenia also demonstrated increased concentrations of KYN (56–65 nM) and KYNA (1.8–2.3 nM) in their cerebrospinal fluid, compared to KYN (27–30 nM) and KYNA (1.3–1.4 nM) concentrations from healthy subjects [5]. Therefore, KYN and its metabolites may represent potential biomarkers for healthcare professionals to provide diagnosis and prognosis.

A biosensor is a transformative tool used for the measurement of a substance of interest. The main principle is based on biosensing by a bioreceptor to the targeted substance, which is then transduced by biological signals to emit quantifiable and interpretable signals [6]. Biosensors can be classified into several categories based on detection systems, transducers, technology and bioreceptors [7]. The common classification is based on the biosensing method and signal transduction perspectives, including catalytic enzyme reactions, protein receptor sensing, antigen–antibody interactions, aptamer-based recognition and whole-cell-based detection [8]. Biosensors can also be classified based on the mode of signal transduction, which depends on the operational methods of the transducer in converting these biosensing events into detectable signals, such as optical, electrochemical, thermal or gravimetric [6,7].

A microbial biosensor is a whole-cell-based biosensor, employing micro-organisms such as bacteria, fungi, protozoa or viruses as the biosensing element [7,9]. The biosensing process in a microbial biosensor occurs through the physiologic or metabolic responses of microbes upon exposure to the targeted substance [9]. For instance, a whole-cell-based *Escherichia coli* biosensor was constructed to detect heavy metals by engineering the bacteria with a metal-sensitive plasmid. Upon sensing the target metal, the promoter of the metal-sensitive gene (i.e. the *zntA* gene) is activated, driving the expression of the reporter gene (i.e. the *lux* gene), which then emits an optical signal [10]. Microbial biosensors offer several advantages, including robust production of different modifications, a broad range of substance detection and ease of genetic engineering to achieve optimal effectiveness [9,11]. *E. coli* is one of the most widely used chassis for microbial biosensors due to its well-defined genetic profiles, rapid proliferation rate and suitability for genetic modification [12]. An example of the adoption of *E. coli*-based biosensors is the detection of bile salts in serum samples from hepatic transplant patients. This biosensor model employed the TcpH/TcpP regulatory system in *Vibrio cholerae* for bile salt sensing, which was integrated into *E. coli* to enable the detection of bile salts, a biomarker for liver dysfunction [13]. This sheds light on promising avenues for developing cost-effective biomedical devices for biomarker screening and diagnostic testing.

**Fig. 1.**
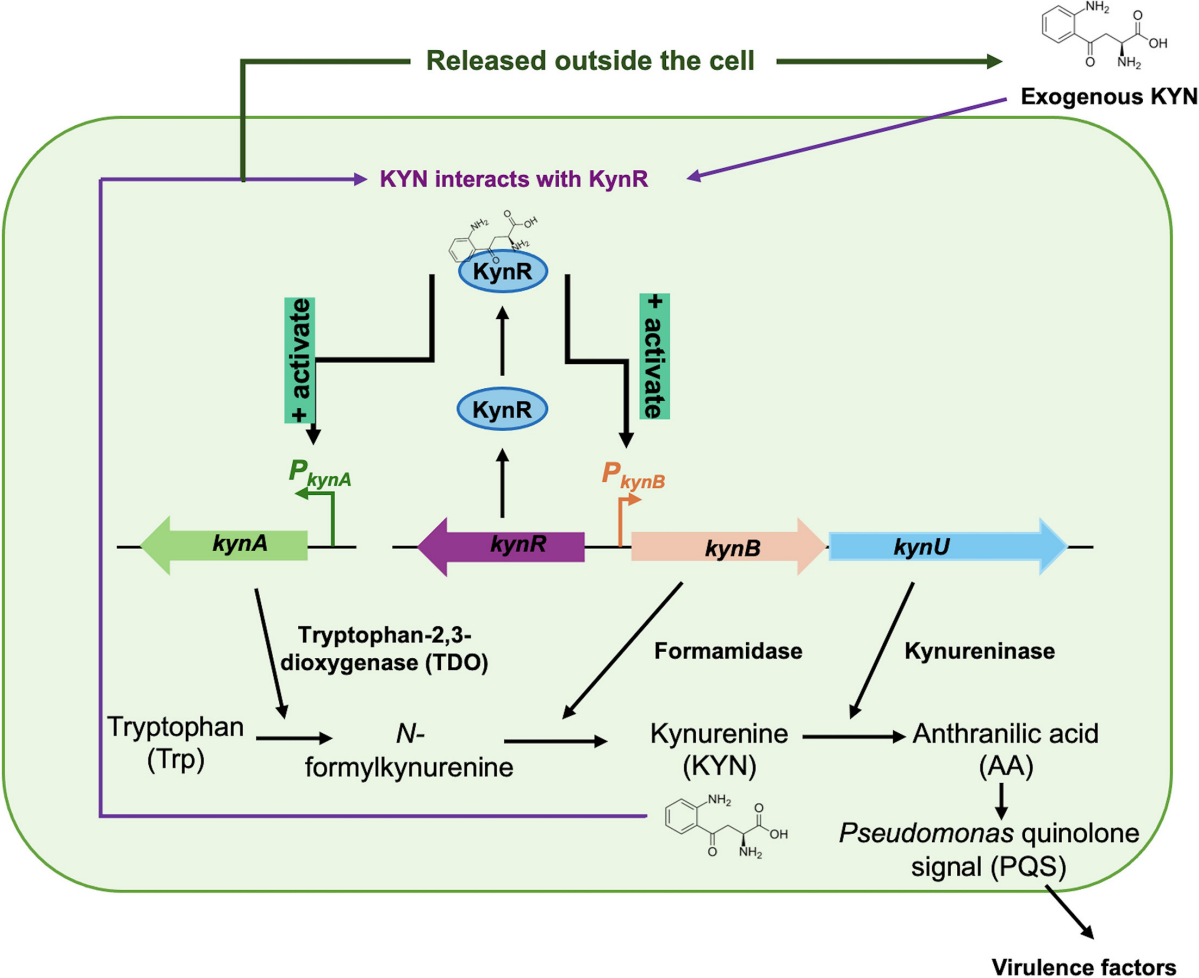
KYN regulon consists of *kynR*, *kynA*, *kynB* and *kynU*, which encode KynR, TDO, formamidase and kynureninase, respectively. KynR detects the exogenous KYN and subsequently activates the *kynA* and *kynB* promoters, inducing the expression of these enzymes responsible for KYN metabolism in *P. aeruginosa*, consequently leading to the production of PQS.

The current standard method for KYN detection in the biomedical field relies on expensive analytic chemistry methods, e.g. mass spectrometry (MS), which is highly technically demanding and time-consuming. The development of a microbial biosensor may present an alternative option for rapid and convenient detection. Interestingly, only a few bacterial families, such as *Pseudomonadaceae* and *Bacillaceae*, possess their own KYN pathway [14]. Among these bacteria, the KYN pathway in *Pseudomonas aeruginosa* is the most recognized and characterized bacterial KYN pathway. The KYN regulon in *P. aeruginosa* contains the *kynR* gene that encodes the KYN pathway regulator (KynR), which serves a crucial role in sensing exogenous KYN. In the presence of KYN, KynR can activate the expression of the downstream gene *kynB*, which encodes a formamidase. However, the role of KYN in the activation of *kynA*, which encodes TDO, remains unclear, as the *kynA* promoter exhibits constitutive activity in a *kynA′-lacZ* transcriptional fusion. Interestingly, KynR binds to the *kynA* promoter only in the presence of KYN (Fig. 1) [15]. Transcription of these enzymes contributes to bacterial virulence by promoting *Pseudomonas* quinolone signal (PQS) production by converting KYN into AA via kynureninase, allowing AA in turn to be converted into PQS. Therefore, the enzymes TDO, formamidase and kynureninase are essential for the KYN pathway in *P. aeruginosa* and PQS production and are orchestrated by KynR [14].

In this study, we integrated *P. aeruginosa* KynR regulon into KYN detecting modules with fluorescent output in *E. coli* chassis as a biosensor for KYN detection. Despite several attempts with different designs and verification, biosensors failed to produce a significant fluorescence signal in the presence of physiological levels of KYN, suggesting the sensitivity of * P. aeruginosa* KynR is insufficient and KynR may not function in the *E. coli* chassis. Further exploration of alternative KYN sensing and responding components is needed to develop convenient biosensors for KYN sensing.

## Methods

### Plasmid designs

Our pilot biosensors were pMA-KYN and pMK-KYN. The pMA-KYN biosensor is an *E. coli* containing plasmid designed to detect exogenous KYN and transmit a fluorescence signal. The plasmid comprises *kynR*, the *kynA* promoter (*P_kynA_*) and *gfp*, with a constitutively expressed promoter *ProD* to drive *kynR* expression. The pMK-KYN biosensor was modified from the pMA-KYN biosensor with modifications, including the substitution of the *kynA* promoter with the *kynB* promoter (*P_kynB_*) and replacement of *gfpmut3* as the reporter gene (National Center for Biotechnology Information[NCBI] GenBank: ABF74540.1), which encodes the green fluorescence protein (GFPmut3). The *kynR* gene, *kynA* promoter and *kynB* promoter sequences were derived from * P. aeruginosa* PA14 [16]. The selected promoter regions of *kynA* (−208 bp to+20 bp relative to the ATG of *kynA*) and *kynB* (−289 bp to+89 bp relative to the ATG of *kynB*) were incorporated into the designs, and their binding to the KynR was confirmed by the electrophoretic mobility shift assay [15].

**Fig. 2.**
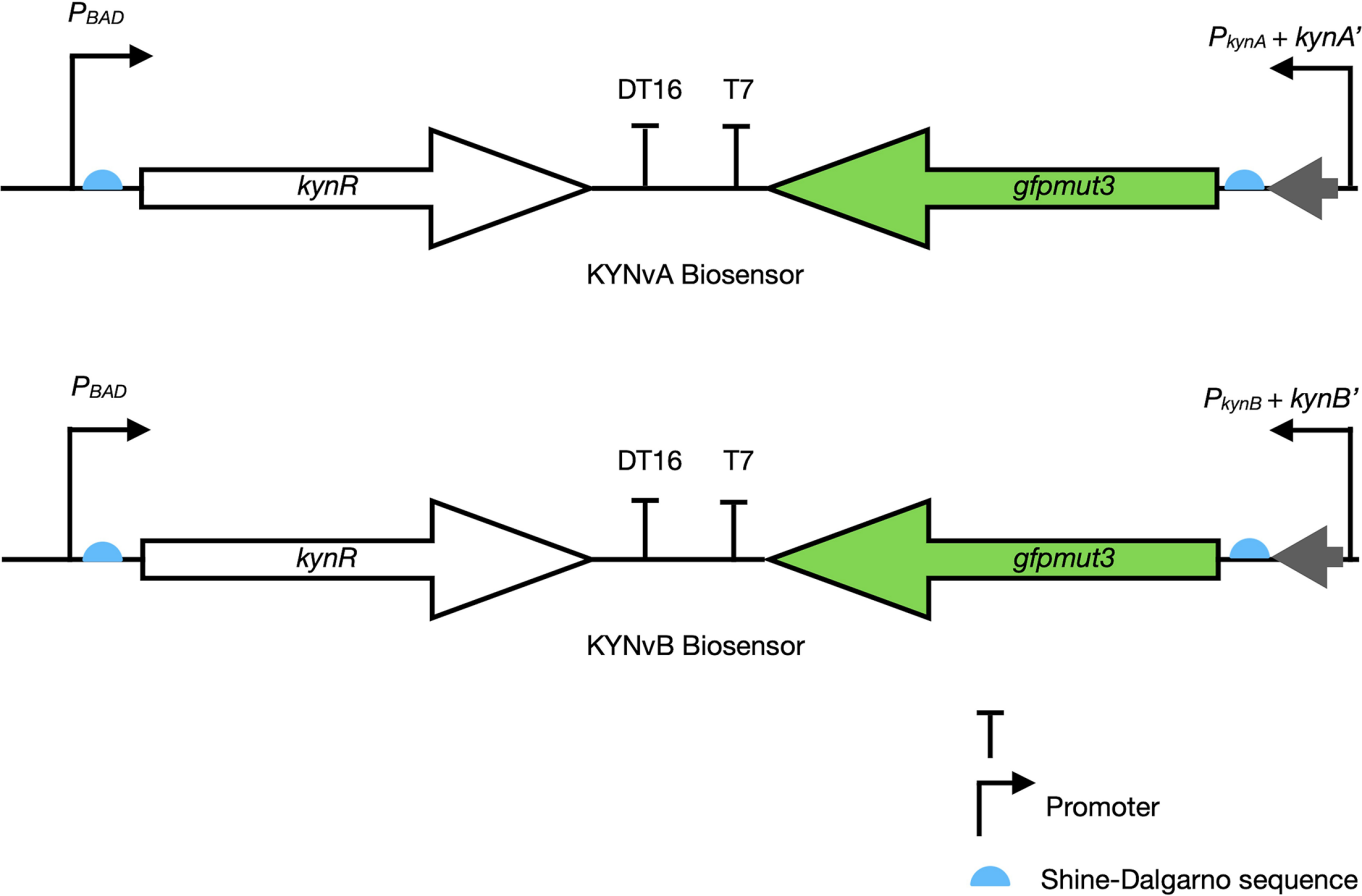
KYNvA (top) and KYNvB (bottom) biosensors employed the *P_BAD_* promoter to induce the *kynR* expression through arabinose induction. KynR would sense the exogenous KYN and activate the *kynA* or *kynB* promoter to drive the expression of the reporter gene to produce the fluorescence signals.

To ensure the expression of *kynR*, the replacement of the constitutive *ProD* promoter with the inducible *P_BAD_* promoter was performed. The developed plasmid designs utilized for KYN detection in this study were categorized into two versions based on their promoters: KYNvA, consisting of the *kynA* promoter, and KYNvB, consisting of the *kynB* promoter (Fig. 2). Both versions contain their truncated downstream gene ORFs (20 bp in the 5′ end for *kynA* and 89 bp in the 5′ end for *kynB*) to ensure the native promoters remain intact for KynR binding. These promoters were engineered with *gfpmut3*. The biosensor operation hinges on the promoter *P_BAD_*, which is inducible by arabinose. The arabinose induction would enable *kynR* expression, allowing KynR to sense exogenous KYN, subsequently activating either the *P_kynA_-gfpmut3* or *P_kynB_-gfpmut3* gene to produce a fluorescence signal. Strong DT16 and T7 transcriptional terminators [17] and Shine–Dalgarno sequence (AGGAGG) for *E. coli* ribosome binding are also integrated into the biosensor designs (Fig. 2). Genetic modules were synthesized, cloned into pBR322*ori* high-copy number plasmid pBAD24 and transformed into *E. coli* DH10B (Thermo Fisher Scientific, Waltham, MA, US). Plasmid sequences are available in Material S1, available in the online Supplementary Material.

### Culture medium and conditions

The pilot biosensors were cultured overnight on Luria–Bertani (LB) agar supplemented with ampicillin 100 µg⋅ml^−1^ for pMA-KYN and kanamycin 50 µg⋅ml^−1^ for pMK-KYN at 37 °C. A fresh colony was inoculated into LB broth supplemented with ampicillin 100 µg⋅ml^−1^ for pMA-KYN and kanamycin 50 µg⋅ml^−1^ for pMK-KYN and incubated at 37 °C with shaking at a speed of 200 r.p.m.

KYNvA and KYNvB were cultured on LB agar plates supplemented with carbenicillin 50 µg⋅ml^−1^ and incubated overnight at 37 °C. A fresh colony was inoculated into LB broth supplemented with carbenicillin 50 µg⋅ml^−1^ and incubated overnight at 37 °C with a shaking speed of 200 r.p.m. before further experimentation.

### RNA extraction and reverse transcription PCR 

The overnight culture of KYNvA and KYNvB was subcultured to an optical density at a wavelength of 600 nm (OD_600_) of around 0.06–0.08 and incubated for a further 2 h until it reached OD_600_ of 0.3–0.8. The culture was split into three groups: 1% (w/v) arabinose (Thermo Fisher Scientific) induction, 0.5% (w/v) arabinose induction and no arabinose induction, followed by a further incubation at 37 °C for 2 h. Bacterial cells were harvested by centrifugation at 3,800 ***g***, and the supernatant was discarded. The pellet was resuspended in 120 µl spheroplasting lysis buffer (spheroplasting/26% (w/v) raffinose buffer, 1 mg⋅ml^−1^ lysosyme, 250 U mutanolysin) and incubated at 37 °C for 5 min. The lysate was transferred to a screw-cap tube containing 250 µl iced zirconia beads (Thermo Fisher Scientific) and homogenized using TissueLyser LT (QIAGEN, Hilden, Germany) at 50 Hz for 6 min. After homogenization, the lysate was centrifuged at 12,000 ***g*** for 1 min, followed by the addition of 350 µl PureLink™ lysis buffer (Thermo Fisher Scientific). RNA purification was then performed according to the manufacturer’s protocol for the PureLink^TM^ RNA Mini Kit (Thermo Fisher Scientific).

High-Capacity cDNA Reverse Transcription Kit (Thermo Fisher Scientific) was employed to conduct reverse transcription PCR according to the manufacturer’s protocol. The primers used for *kynR* amplification were as follows: forward, CCATGGTACATGCCCCTGGAC, reverse, GCAGGTCGACTCTAGATCAACTCTTCAGG, with a 477 bp product. The reverse transcription PCR products were examined by 1% (w/v) agarose gel electrophoresis at 85V for 40 min to confirm the expression of *kynR* RNA.

### Biosensor characterization

Following overnight incubation of the pilot biosensors, the culture was subcultured to an OD_600_ of 0.05 (OD_490_ of 0.1 for pMA-KYN). The experiment was divided into five groups, each receiving 1, 5, 10 or 100 µM of KYN (Sigma-Aldrich, Darmstadt, Germany) or no KYN in a black micro clear 96-well plate (Greiner Bio-One, Stonehouse, UK).

The overnight culture of KYNvA and KYNvB was subcultured and incubated until reaching an OD_600_ of 0.3–0.8 before arabinose induction. The experiment was divided into three groups, consisting of 1% (w/v) arabinose induction, 0.5% (w/v) arabinose induction and no arabinose induction. They were then further incubated at 37 °C for an additional 2 h. Subsequently, each group was divided into two subgroups: one subgroup received 1 mM KYN addition, while the other subgroup did not receive KYN. In a black micro clear 96-well plate, 200 µl of each subgroup was added per well in triplicate. The LB broth alone was used as the negative control, while *E. coli* DH5α harbouring the plasmid pUC18T-mini-Tn7T-Tp-gfpmut3 (NCBI GeneBank: DQ493881) served as the positive control for fluorescence detection [18].

The OD absorbance and fluorescence were measured at excitation and emission wavelengths of 485 nm and 528 nm, respectively, at 0, 2 and 24 h using the Biotek Synergy HT Microplate Reader (Marshall Scientific, Hampton, NH, USA). The experiments were conducted in three separate biological trials.

### Fluorescence microscopy

Biosensor strains (pMK-KYN, KYNvA and KYNvB) were cultured for 24 h under the conditions described above. Following incubation, 10 µl of each culture was mounted on a glass slide and covered with a coverslip. Samples were imaged using a fluorescence microscope at 60× magnification. Brightfield and green fluorescence images (excitation/emission at 488/510 nm) were captured. The *pUC18T-mini-Tn7T-Tp-gfpmut3* strain was used as a positive control for GFP expression.

### Statistical analysis

Data obtained from the biosensor characterization were recorded in Microsoft Excel 16 (Microsoft, Redmond, WA, USA) for subsequent analysis. Graph generation and statistical analysis were performed using GraphPad Prism 10 (GraphPad Software Inc., La Jolla, CA, USA). The one-way ANOVA was conducted for comparison among the groups. The independent t-test was used for comparison between groups, with a *P*-value less than 0.05 considered statistically significant.

## Results

### Pilot biosensors

**Fig. 3.**
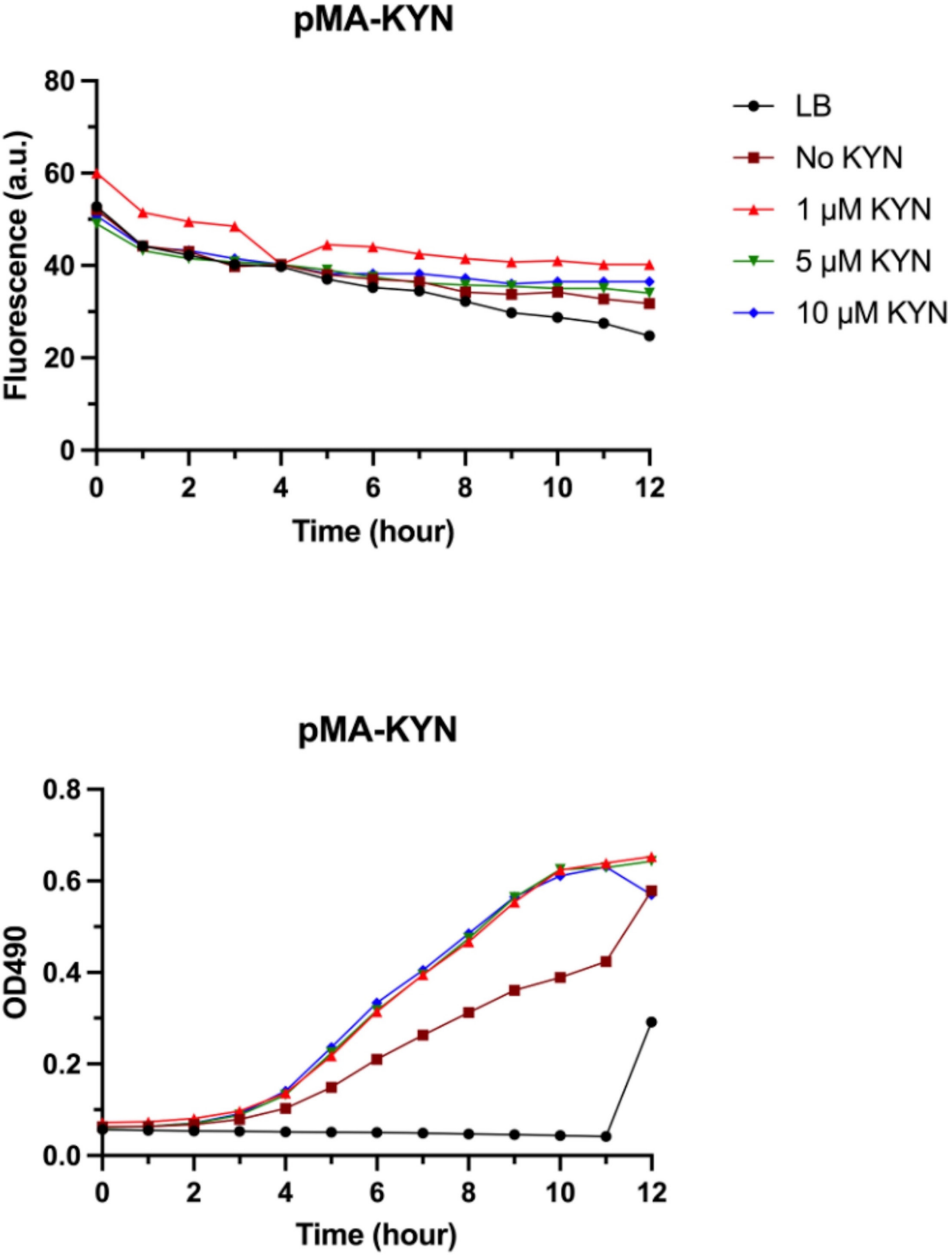
Fluorescence produced by the pilot biosensor, pMA-KYN, in conditions of no KYN, 1 µM, 5 µM, 10 µM KYN and LB, which served as the negative control (top). There were no remarkable fluorescence signals observed from any group, despite the increase in bacteria numbers over time (bottom).

**Fig. 4.**
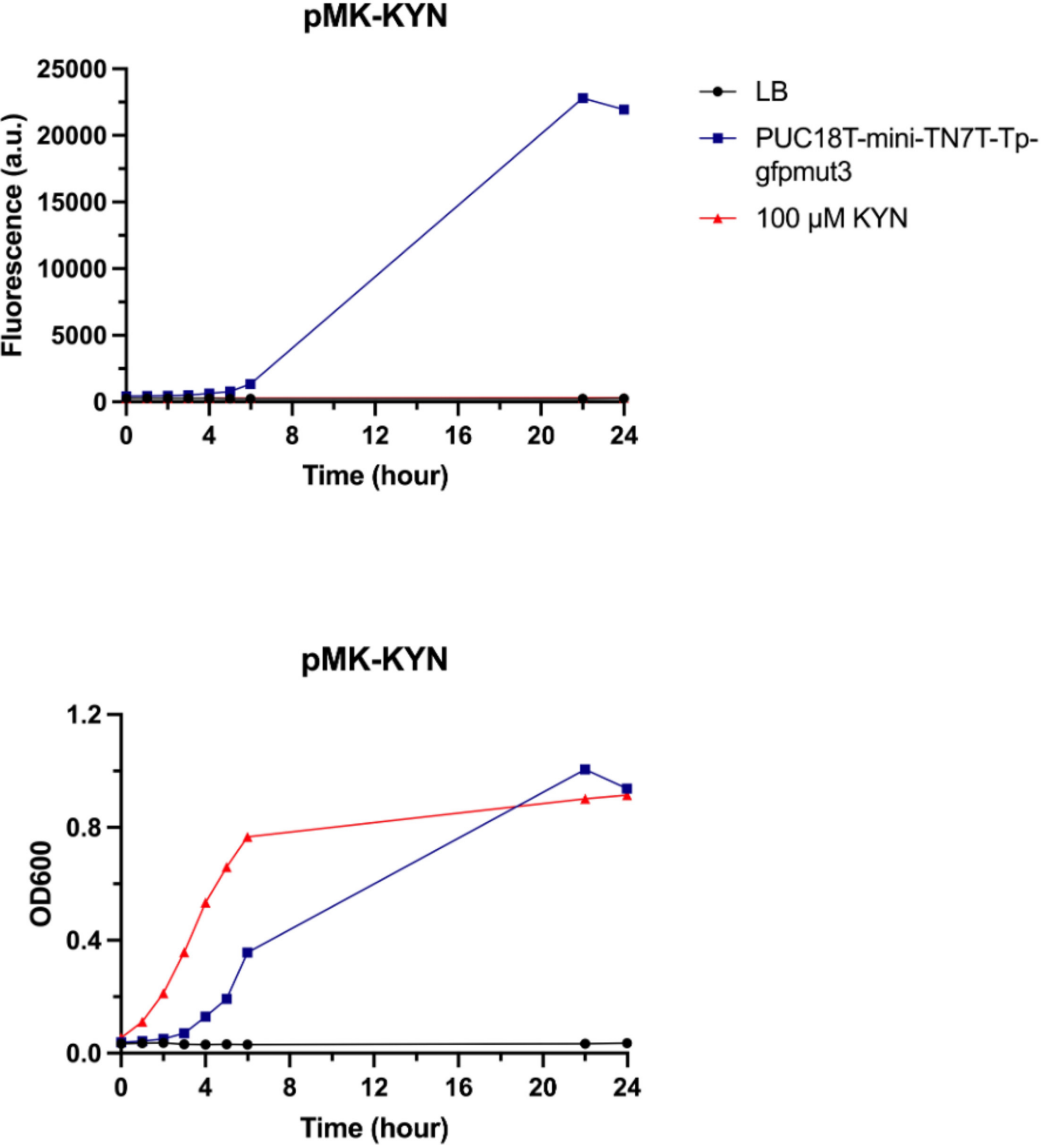
The pilot biosensor, pMK-KYN, was the developed variant of the pMA-KYN biosensor, which exhibited growth over time (bottom). However, it failed to produce a remarkable fluorescence signal as anticipated, in contrast to pUC18T-mini-TN7T-Tp-gfpmut3, which served as a positive control, showing a strong fluorescence signal from 5 to 24 h (top).

The results obtained from pMA-KYN did not show any discernible fluorescence signal after 12 h in the 1, 5 or 10 µM KYN groups. The transmitted signals exhibited similarity to those observed in the group lacking KYN addition (Fig. 3). Similarly, the pMK-KYN biosensor did not show a remarkable fluorescence signal upon addition of 100 µM KYN over a 24-h period (Fig. 4). The pUC18T-mini-Tn7T-Tp-gfpmut3 was used as fluorescence production control and demonstrated a stronger signal compared to the pMK-KYN biosensor from 5 to 24 h. Raw data of bacterial growth and fluorescence production are available in Material S2. It is important to note that M9 minimal medium was attempted to use as an alternative to LB medium to reduce background noise and to improve the signal-to-noise ratio for fluorescent detection; however, the pilot biosensor showed a similar fluorescence signal when compared to M9 minimal medium alone, which has been provided in Material S2. Additionally, the *ProD* promoter failed to drive *kynR* expression as indicated by reverse transcription PCR (Fig. S1).

### *kynR* expression under arabinose induction

**Fig. 5.**
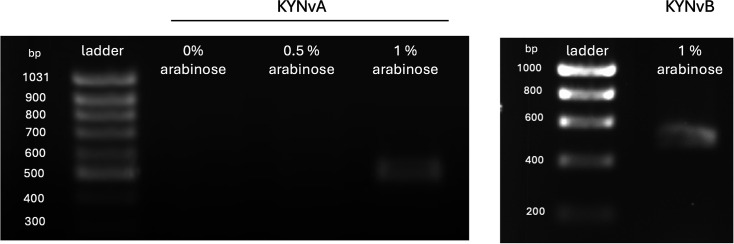
The *kynR* expression was confirmed by reverse transcription PCR, as indicated by a distinct band (477 bp). The *kynR* expression was successfully induced by 1% (w/v) arabinose for both KYNvA (left) and KYNvB (right). In contrast, no observable bands were present under 0.5% (w/v) arabinose or in the absence of arabinose induction.

Due to the lack of KynR expression under the *ProD* promoter, the *P_BAD_*-driven system was adopted and incorporated into KYNvA and KYNvB, enabling tighter control and inducible expression for systematic evaluation. To determine the optimal arabinose concentration for inducing the *P_BAD_* promoter to drive *kynR* expression in KYNvA and KYNvB biosensors, three conditions were examined: no arabinose, 0.5% (w/v) arabinose and 1% (w/v) arabinose. The reverse transcription PCR products were analysed on agarose gel electrophoresis and showed that only 1% (w/v) arabinose successfully induced *P_BAD_* to drive *kynR* expression, as illustrated by the presence of a distinct band corresponding to the expected size (477 bp) (Fig. 5). Conversely, 0.5% (w/v) arabinose failed to induce the *P_BAD_* promoter, showing no observable band similar to the condition without arabinose induction (Fig. 5).

### Functional characterization to detect KYN

**Fig. 6.**
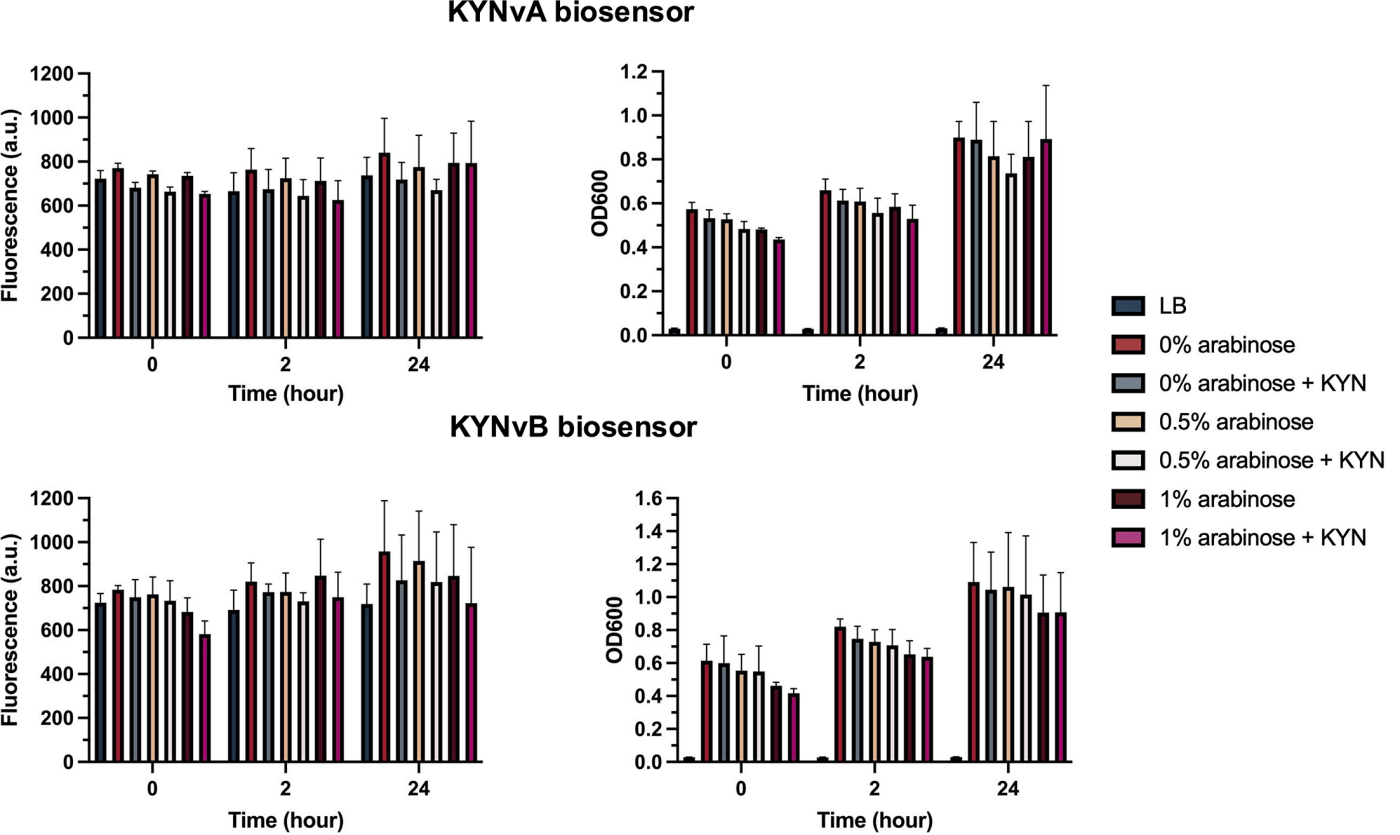
Despite the bacterial growth, KYNvA (top) and KYNvB (bottom) failed to generate significant fluorescence signals upon the induction of 1% (w/v) arabinose and the addition of 1 mM KYN. There was no significant difference in fluorescence intensities compared to the groups lacking KYN in the 24 h period (KYNvA, *P*=0.99; KYNvB, *P*=0.78). No significant differences in fluorescence production were observed among groups at 24 h (KYNvA, *P*=0.73, KYNvB, *P*=0.79).

KYNvA and KYNvB biosensors were employed to detect the exogenous KYN at a concentration of 1 mM under varying levels of arabinose induction (0, 0.5 and 1%). Bacterial cell growth was monitored by measuring OD_600_ to ensure that fluorescence production was not affected by impaired growth (Fig. 6). Despite arabinose induction and confirmed bacterial growth, no remarkable fluorescence signal was detected in either biosensor in a 24-h period (Fig. 6). A one-way ANOVA revealed no significant differences in fluorescence production among groups at 24 h for KYNvA, *F* (6, 14) = 0.5, *P*=0.73, and KYNvB, *F* (6, 14) = 0.5, *P*=0.79. Moreover, the fluorescence signals observed were comparable to those of the control groups without KYN addition at 24 h for KYNvA with 1% (w/v) arabinose induction, *t* (4) = 0.01, *P*=0.99, and KYNvB with 1% (w/v) arabinose induction, *t* (4) = 0.30, *P*=0.78 (Fig. 6).

**Fig. 7.**
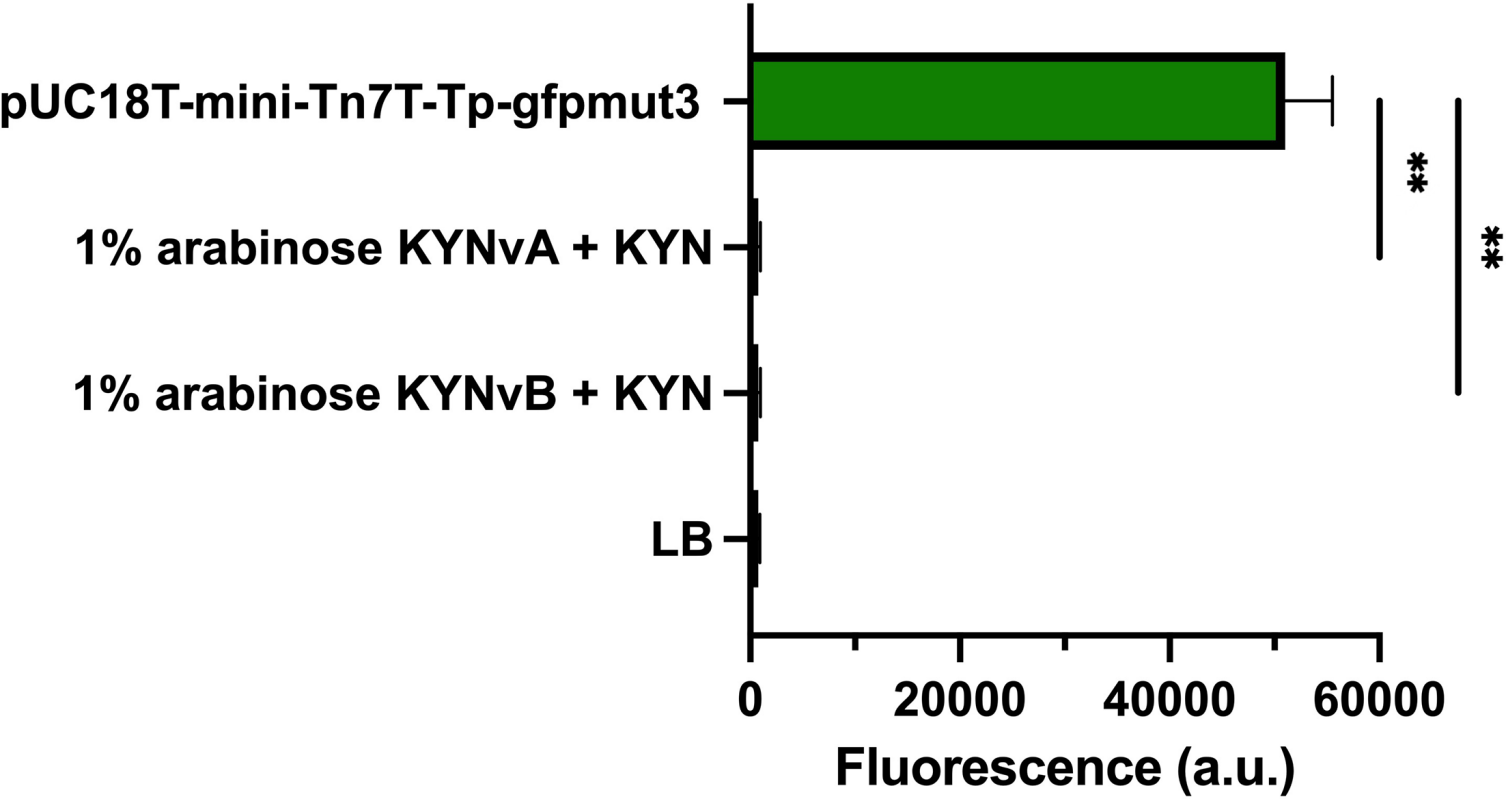
*E. coli* pUC18T-mini-Tn7T-Tp-gfpmut3 served as the positive control, exhibiting significantly higher fluorescence intensity than KYNvA (*P*=0.0026) and KYNvB (*P*=0.0026) under 1% (w/v) arabinose and 1 mM KYN at 24 h. LB was used as the negative control, displaying fluorescence intensity comparable to KYNvA (*P*=0.91) and KYNvB (*P*=0.87).

To verify the fluorescence signal, a positive control, *E. coli* pUC18T-mini-Tn7T-Tp-gfpmut3, which harbours a constitutive *gfpmut3,* demonstrated a high intensity of fluorescence signal at 24 h without arabinose (*M*=51,036, sd=4,507). However, KYNvA and KYNvB with 1% (w/v) arabinose and 1 mM KYN exhibited significantly lower fluorescence signals compared to *E. coli* pUC18T-mini-Tn7T-Tp-gfpmut3 at 24 h, with KYNvA: *t* (4) = 19.29, *P*=0.0026; KYNvB: *t* (4) = 19.29, *P*=0.0026 (Fig. 7). Furthermore, the fluorescence intensities of KYNvA and KYNvB did not differ significantly from the LB control at 24 h, with KYNvA: *t* (7) = 0.12, *P*=0.91; KYNvB: *t* (7) = 0.17, *P*=0.87 (Fig. 7). These findings indicate that neither KYNvA nor KYNvB functioned as presumed even in the presence of 1 mM KYN and 1% (w/v) arabinose induction, with no observable differences compared to conditions without KYN and LB alone. Raw data of bacterial growth and fluorescence production are available in Material S2.

## Discussion

The biosensor design in this study utilized the *kynR* gene from the KYN regulon of *P. aeruginosa* as the biological sensing component for the selective detection of exogenous KYN. KynR acts as a crucial transcriptional regulator for the KYN pathway in *P. aeruginosa*, upregulating the expression of genes *kynA*, *kynB* and *kynU* in response to KYN, which encode TDO, formamidase and kynureninase, respectively [14,15]. The transcription of *kynA* and *kynB* genes was demonstrated to be regulated dependently by KynR [15]. Additionally, KYN serves as a co-inducer, enabling KynR to bind to the *kynA* promoter to activate gene expression. On the contrary, the *kynB* promoter does not require KYN for activation, as it was shown that KynR can bind to the *kynB* promoter in the absence of KYN [15]. Therefore, our study developed biosensors based on the current available knowledge of the bacterial KYN regulatory mechanisms to examine whether KYNvA, KYNvB or both can produce a detectable signal upon the presence of KYN.

The pilot biosensors were designed to operate under the constitutive expression of the *ProD* promoter. Both pMA-KYN and pMK-KYN failed to produce strong fluorescence signals. The biosensor cells from pMK-KYN were also directly observed under fluorescence microscopy; however, they did not exhibit any detectable fluorescence signal compared to pUC18T-mini-Tn7T-Tp-gfpmut3 (Fig. S2). Moreover, reverse transcription PCR was performed to confirm the expression of *kynR*, indicating that the constitutive *ProD* promoter failed to drive *kynR* expression (Fig. S1). Therefore, the biosensor was developed for KYNvA and KYNvB using the inducible *P_BAD_* promoter to activate *kynR* expression. The *P_BAD_* promoter was selected for its tight regulation by arabinose, remaining repressed in the absence of arabinose and strongly activated when arabinose is present [19]. Additionally, the *P_BAD_* promoter has been shown to function effectively even in the presence of glucose, as demonstrated in a previous study [20]. Our results demonstrated that *kynR* expression was not activated without arabinose induction or 0.5% (w/v) arabinose, as no expected band was observed from the reverse transcription PCR products (Fig. 5). However, in the presence of 1% (w/v) arabinose, it can successfully induce the *P_BAD_* promoter, driving *kynR* expression in both KYNvA and KYNvB, as indicated by the distinct bands from the reverse transcription PCR products (Fig. 5). Given that *E. coli* is a well-characterized model organism in which transcription and translation are generally well-coupled and efficient, the likelihood of poor translation or protein instability of KynR is considered relatively low.

In our pilot biosensor model, KYN at concentrations of 1, 5, 10 and 100 µM was examined to characterize the function of the biosensor. However, the pilot biosensor failed to produce a strong fluorescence signal in response to these concentrations. Following the unsuccessful results obtained from the pilot biosensor, the current version of biosensors, KYNvA and KYNvB, was developed. To enhance responsiveness, the highest tested concentration of 1 mM KYN was used for functional characterization. Unfortunately, even with this high KYN concentration at 1 mM, both KYNvA and KYNvB exhibited fluorescence signals similar to the control groups without KYN addition (Fig. 6). Moreover, these biosensors were confirmed to have a negligible amount of fluorescence production through comparison with *E. coli* pUC18T-mini-Tn7T-Tp-gfpmut3, revealing that KYNvA and KYNvB produced significantly lower fluorescence signals (Fig. 7). These KYNvA and KYNvB biosensors also lacked visible fluorescence when observed directly under a fluorescence microscope (Fig. S2).

Although KynR binding to the *kynA* and *kynB* promoters was confirmed in gel shift assays [15], the results suggest that KynR from *P. aeruginosa* may not function properly to detect KYN in the *E. coli* chassis, resulting in insignificant fluorescence signals in KYNvA and KYNvB. In contrast, Knoten and coworkers demonstrated that 1 mM KYN could induce the expression of *lacZ* in the KynR expression plasmid in *E. coli*, as measured by *β*-galactosidase activity [15]. While LacZ-based reporter benefits from enzymatic amplification, they are less practical for rapid and convenient detection. To develop a versatile biosensor suitable for biomedical applications, a GFP-based biosensor was employed in this study due to its advantage for real-time monitoring. Nevertheless, the weak fluorescence signals in KYNvA and KYNvB may be attributed to GFP relying on proper protein folding and chromophore maturation, which may result in low sensitivity to detect weak transcriptional activation. However, the absence of fluorescence under direct microscopic observations suggests that this is unlikely due to GFP instability or degradation. Given these results, other upstream factors such as suboptimal KynR activity may contribute to limiting biosensor functionality.

It is important to note that physiological KYN concentrations are considerably lower than those concentrations used in laboratory experiments, typically ranging from 1.3 to 2.4 µM in human plasma and serum [21]. In healthy individuals, KYN concentrations in saliva were even lower, ~0.04–0.08 µM [22]. Furthermore, in inflammatory conditions such as the acute phase of the coronavirus disease 2019 (COVID-19), serum KYN concentrations were significantly increased; however, the concentrations were around 2.0–19.6 µM [23]. The KYN concentrations considered useful as a prognostic biomarker in patients with hepatocellular carcinoma were also relatively low, ~2.6 µM [4].

This information suggests that a biosensor intended for KYN detection in clinical settings would require a minimum detection threshold in the micromolar range. The biosensors in this study were developed aiming for screening KYN levels with high sensitivity and rapid detection capabilities for biomedical applications. Therefore, the biosensor based on *P. aeruginosa* KynR may not be fully functional in an *E. coli* background, which results from a lack of the key *Pseudomonas* factors or chaperones to facilitate KYN sensing by KynR. Furthermore, the extremely low fluorescence signals observed across all biosensors in this study suggest that the current *E. coli*-based biosensor lacks sufficient sensitivity to detect physiologically relevant KYN levels and generate a robust signal. Additionally, while *E. coli* lacks its own KYN pathway, Han and coworkers demonstrated that aspartate aminotransferase, a protein isolated from *E. coli*, exhibits KAT activity, enabling the catabolism of KYN to KYNA [24]. This enzymatic activity may interfere with KynR sensing. Our study also suggests the complexity of the KYN regulatory mechanism in *P. aeruginosa*, which may require additional investigation. To develop more effective biosensors for this purpose, further exploration of alternative biological sensing components is warranted. Alternatively, the biomimetic liposomes containing key sensing factors and components from *Pseudomonas* may provide a convenient biosensing approach without bacterial virulence concern.

## Supplementary material

10.1099/acmi.0.001031.v5 Uncited Supplementary Material 1.

10.1099/acmi.0.001031.v5 Uncited Supplementary Material 2.

## Abbreviations

AA: anthranilic acid
KYN: kynurenine
KYNA: kynurenic acid
KynR: KYN pathway regulator
LB: Luria–Bertani
PQS: *Pseudomonas* quinolone signal
QA: quinolinic acid
TDO: tryptophan 2,3-dioxygenase
Trp: tryptophan
