# Supplementary material for: Functional characterization of engineered bacterial biosensors for kynurenine detection
Source: Access Microbiol. 2025 Aug 8;7(8):001031.v5. doi: 10.1099/acmi.0.001031.v5 (PMC12451312; doi:10.1099/acmi.0.001031.v5)
Supplement: Uncited Supplementary Material 1. [file acmi-7-01031-s001.pdf]

Supplementary Material

Functional Characterisation of Engineered Bacterial Biosensors for Kynurenine Detection

Pisit Charoenwongwatthana[1,2], Halah Ahmed[1], Wojciech Cajdler[1], Jamie Coulter[1], Chien-Yi Chang[1]*

[1]School of Dental Sciences, Faculty of Medical Sciences Newcastle University, Framlington Place, Newcastle upon Tyne, NE2 4BW, UK.
[2]Department of Oral Medicine and Periodontology, Faculty of Dentistry, Mahidol University, Bangkok, Thailand.

***Correspondence:** chienyi.chang@newcastle.ac.uk

Keywords: Biosensor, Tryptophan, Synthetic biology, Bioengineering, Kynurenine

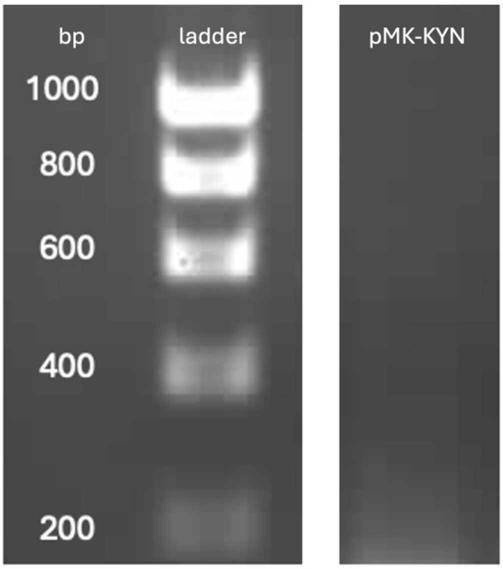

Supplementary Fig. 1
The *ProD* promoter failed to drive kynR expression in the pMK-KYN biosensor, as indicated by the absence of the observable *kynR* band (477 bp) in the RT-PCR products.

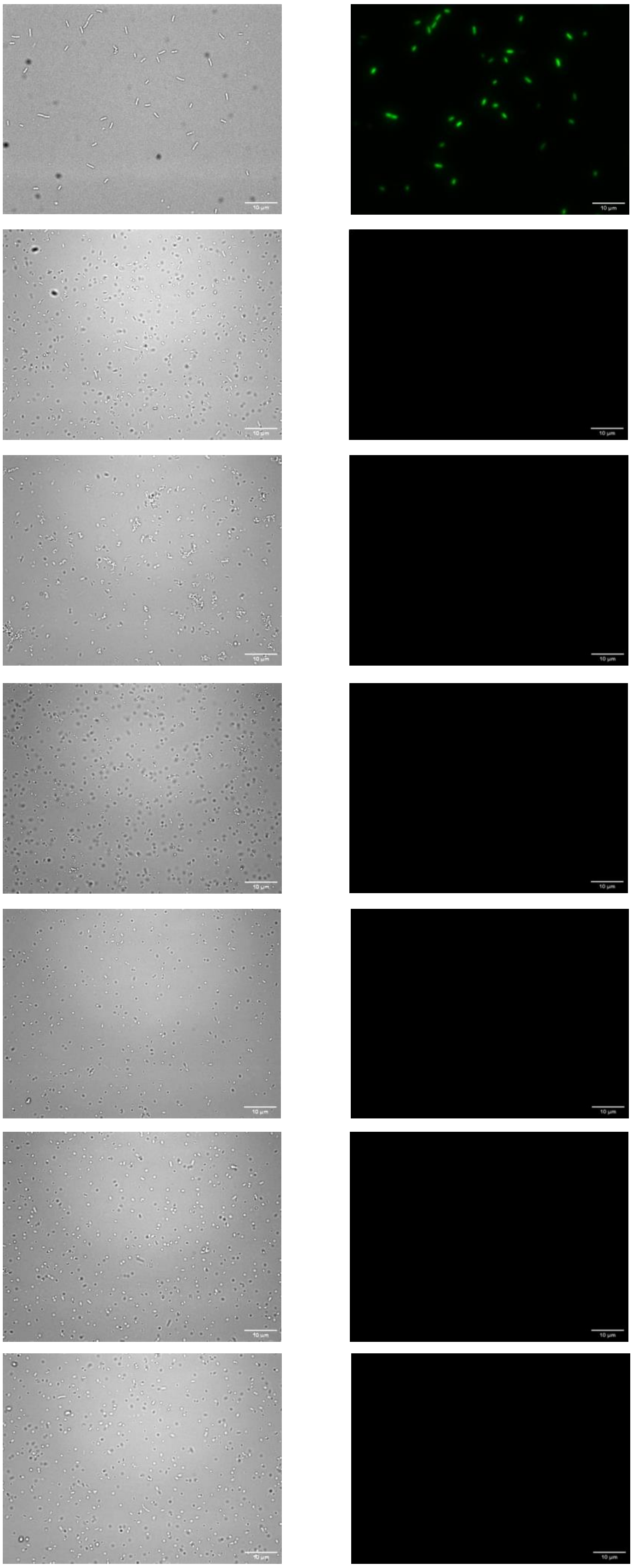

pUC18T-mini-Tn7T-Tp-gfpmut3

pMK-KYN

pMK-KYN with KYN

KYNvA

KYNvA with KYN

KYNvB

KYNvB with KYN

Supplementary Fig. 2

Biosensor cells from the pMK-KYN, KYNvA, and KYNvB constructs were cultured according to the methods described in the main text. After 24 hours of growth, 10 μL of each culture was mounted on a glass slide and examined under a fluorescence microscope at 60× magnification. Brightfield imaging confirmed the presence of bacterial cells in all conditions (left panels). Only the positive control strain, pUC18T-mini-Tn7T-Tp-gfpmut3, exhibited detectable fluorescence signals. None of the biosensor strains, with or without KYN supplementation, displayed visible fluorescence when excited at 488 nm (right panels). Scales bars are 10 μm.

Bio-components sequences

Part name	DNA sequence	Ref.
kynR	ATGCCCCTGGACGCCATCGATCTGCGCATTCTCCGTCACCTGCAGCAGGACGG<br>GCGGATGAGCAACCAGGACCTGGCGGAGAGGGTTTCCCTCTCGCCATCCGCCT<br>GCCTGCGCCGGCTGCGCCTGCTGGAAAGCGAGGGGATCATCCGCGGCTACTCG<br>GTGGAGCTGGATGCCGAGCGTCTGGGCGTGGAGCTGGAAGCCATCGTCCATGT<br>GTCGCTGCGCCAGGACGTCGAGGGTTGGCACGAGGCGTTCATCGCCAGGGTCC<br>GGGACTGGCCGGAGGTGGTCACCGCCTATGTGGTGACCGGCGCCACCAACTAT<br>GTGCTACGGGTCCGGGCGCGCAACCTCAAGCACTATTCGGACTTCATCGTGAA<br>CAAACTGAACCGCGCCGCCGGCGTCACCGATATCCGCTCGGAAATCGTCCTGC<br>AGGAGATCAAGGCCGGTGCCGACCTGCTCGACCTGGTGAACCTGAAGAGTTGA	(1)
kynA	TCGAAGGGAAAGGCGTGGATGCGGTGTATTTCCCCTTCCACAAGGCCAACCAG<br>TTCCTCGGCATGTCCGGCCTGCCGACGTTCCTCGCGGTGGATGTGATGAAGCG<br>CCCGGACGTCCCGGCTGCCGTCGCTGCCTACCAGGCGCACCTGGACAGGGTGT<br>TCGGGCGCGCCGGCTGAGTTCGACGGCGGGCTGCGTGTCGCTTCGCTGGGTGG<br>GGCGGCGGGGAGAGAACCAGGCTCTCGGGACGAGGCCTAACGGTCACGGATCC<br>GCTATCCGGGCTTTTGTCGGCCACGACCCGCGAGTGATCCGAAATTCGATTTT<br>TCCATCGATCCGTCGAATTAGATTTCATCGGGGACGGGGAGGAAAGGATGCTT<br>TGCACGAATATTCGAATGGGCGCATCGTAAAATGAGCGCGCTTCGACCGTGTC<br>GAAGCCACCCCCGACAACGTTGCTGGCAGGTCGCTCATCGACTTCCGACGCCT<br>GCCGCCGAATTCAGGAGATTCACATGTGTCCTTGCCCTCACTCCCAGGCCCAG<br>GGAGAAACGGACGGAGAGGCTGAGTGGCATAACGCCGCCCTGGACTTCACCCA<br>TGCGATGAGCTACGGCGATTACCTGAAGCTGGACAAGGTCCTGGACGCCCAGT<br>TCCCGCTTTCGCCCGACCACAACGAAATGCTGTTCATCATCCAGCACCAGACC<br>TCGGAGCTATGGATGAAGCTGATGCTCCACGAACTGCGGGCCGCCCGGGAGCA<br>CGTCAAGTCAGGCAAGCTCGGTCCGGCGCTGAAAATGCTCGCCAGGGTCTCGC<br>GGATCTTCGACCAGCTGGTACACGCCTGGGCGGTGCTGGCCACCATGACGCCG<br>ACCGAGTACAACACCATCCGCCCGTACCTGGGCCAGTCTTCCGGTTTCCAGTC<br>CTACCAGTACCGCGAGATCGAATTCATCCTCGGCAACAAGAACGCCACCCTGC<br>TCAAGCCCCATGCTCACCGCGCCGAGCTGCTGGCGGCCCTGGAGCAGGCCCTG<br>CATACGCCGTCGCTATACGACGAAGCGATCCGCCTGATGGCCGCCCAGGGGTT<br>GCCGGTAAGCCAGGAGCGGCTCGTCCGGGACGCGGCGGCCGGCACCTGTTACG<br>AGGCCTCGGTGGAGGCGGCGTGGCGACAGGTCTACCAGACGCCGGAGCGCTAC<br>TGGGACCTGTATCAGTTGGCGGAAAAACTGATCGACCTGGAGGATTCGTTCCG<br>CCAGTGGCGCTTCCGTCACGTGACCACGGTGGAGCGCATCATCGGTTTCAAAC<br>CCGGCACGGGCGGAACCGAGGGCGTGGGGTATCTGCGCAGCATGCTCGATACC<br>ATCCTCTTCCCCGAACTGTGGCGGCTCCGTTCCAACCTGTAGTACCGGATGAG<br>GCGCAGCATCCGCCTGCGCCTCAGTCGTTGCCGGCGCCTCCCGCCGCCCACTC<br>TTGCCGCAAAAGCCCCATGAGAACGACATTCCAGCGTTCCCGGCCGACCCGGG<br>TGGCGGAGCGGCGCAAGCCTTCTTCGCGGAACCCCGCGCGTCGATAGAGATGA<br>CGGGCGGCAGCGTTCCAGTCGTACACGTTCAATTCAACGCGCTCGATGTCGGC<br>GTCGGCGAAAGCCTCGGCGAGCAGCGCCTCCAGCATCGGTAGCCCCAGGCCCT<br>GGCCGCGTGCGTTTGGCGCCAGGGCTATGCGAGCCAGGCGGACGACGCCGTTG<br>CGGCGGTCGAACAGCAACTGGCAATGGCCGATGACCTGGCCATCCAGGCAAGC<br>GCTCCAGAGCAGGCGCAGCGGCGGTTGTCGGCGGCTTTCCGCGAGGTCTTCAT<br>GCATCTGCTCGAGGCTCAGCGGATGCCGCAGGGCGGGGCCGGCCCATTGCACC<br>AGCTCCTTTTCC	(1)
kynB	ACGATGAAGTCCGAATAGTGCTTGAGGTTGCGCGCCCGGACCCGTAGCACATA<br>GTTGGTGGCGCCGGTCACCACATAGGCGGTGACCACCTCCGGCCAGTCCCGGA<br>CCCTGGCGATGAACGCCTCGTGCCAACCCTCGACGTCCTGGCGCAGCGACACA<br>TGGACGATGGCTTCCAGCTCCACGCCCAGACGCTCGGCATCCAGCTCCACCGA<br>GTAGCCGCGGATGATCCCCTCGCTTTCCAGCAGGCGCAGCCGGCGCAGGCAGG<br>CGGATGGCGAGAGGGAAACCCTCTCCGCCAGGTCCTGGTTGCTCATCCGCCCG<br>TCCTGCTGCAGGTGACGGAGAATGCGCAGATCGATGGCGTCCAGGGGCATGTG<br>CAATTCCTATCAGAATGAATAGCTATCTTCGAATTTTGTGCGAACAGATTGCC	(1)

	ATGCAGCCTTGCATTTCGCACGAAAATTCTCCGAAATCTCGTCCATTATCATT<br>CGCTGGATTTCCGGAGAAAAACAATGACTTCGCTCCGCTACTGGGACATCAGT<br>CCCGCCCTCGACCCGAGCACGCCGACCTGGCCCGGCGACACGCCGTTCCAGCA<br>GGAATGGGCGGCGCGGCTGGACGAGCAGTGCCCGGTCAACGTCGGCCGGATCA<br>CCCTTTCGCCGCATACCGGCGCCCACGTCGACGGTCCCCTGCACTACCGCGCC<br>GACGGCCTGCCCATCGGCCAGGTGCCGCTGGACATCTACATGGGACCGTGCCG<br>GGTGATCCACTGTATCGGCGCTAACCCGCTGGTGACCCCCGAACACCTCGCCG<br>GCCAGCTCGACGACCTGCCCTCGCGGGTGCTGCTACGGACCTTCGAACGGGTC<br>CCGGCGAACTGGCCGGAAGGCTTCTGCGCTATCGCTCCGGCCACCATCGAGTG<br>CCTCGCCGAGCGCGGCGTCAGGCTGGTCGGCATCGACACCCCGTCGCTCGACC<br>CCCAGCACTCCAAGACCCTCGACGCCCACCACGCGGTGGGCCGCCACGGCATG<br>GCGATCCTCGAAGGCGTGGTCCTCGACGACGTGCCCGCCGGCGACTACGAACT<br>ACTCGCCCTGCCGCTGAAATTCACCCACCTCGATGCCAGCCCAGTGCGTGCCG<br>TGCTGCGCGCCCTGCCTACCGCGGAGTAACCGATGACCACTCGTGACGACTGC<br>CTGGCGCTCGACGCCGGCGATCCCCTGGCCGACCTGCGCCAACTGTTCGCCCT<br>GCCCGACGGCGTGATCTACCTCGACGGCAACTCCCTCGGCGCCCGCCCCCGGG<br>CCGCCGTGGAGCGCGCCGCCGAAGTGGTCGCCGCGGAATGGGGCGAGGGCCTC<br>ATCCGCAGCTGGAACAGCGCCGACTGGCGCGGCCTGCCGGAGCGCCTCGGCGA<br>CAAGCTGGCGCCCCTGATCGGCGCGCGCGCCGGCGAAGTGGTGATCACCGACA<br>CCACCTCGATCAACCTGTTCAAGGTGCTCAGCGCCGCCCTGCGGATCCAGGAG<br>GAAGAAGCGCCAGGGCGCAAGGTGATCGTTTCCGAATCGAGCAACTTCCCCAC<br>TGATCTGTACATCGCCGAGGGCCTTACCGACATGCTCCAGCGCGGCTACCGGC<br>TGCGCCTGGTGGATGGCCCGGAGCAACTGCCGGCGGCGATCGACGCGGATAC	
P_{kynA}-kynA'	CGCGAGTGATCCGAAATTCGATTTTTCCATCGATCCGTCGAATTAGATTTCAT<br>CGGGGACGGGGAGGAAAGGATGCTTTGCACGAATATTCGAATGGGCGCATCGT<br>AAAATGAGCGCGCTTCGACCGTGTCGAAGCCACCCCCGACAACGTTGCTGGCA<br>GGTCGCTCATCGACTTCCGACGCCTGCCGCCGAATTCAGGAGATTCACATGTG<br>TCCTTGCCCTCACTA	(1)
P_{kynB}-kynB'	AGTAGCCGCGGATGATCCCCTCGCTTTCCAGCAGGCGCAGCCGGCGCAGGCAG<br>GCGGATGGCGAGAGGGAAACCCTCTCCGCCAGGTCCTGGTTGCTCATCCGCCC<br>GTCCTGCTGCAGGTGACGGAGAATGCGCAGATCGATGGCGTCCAGGGGCATGT<br>GCAATTCCTATCAGAATGAATAGCTATCTTCGAATTTTGTGCGAACAGATTGC<br>CATGCAGCCTTGCATTTCGCACGAAAATTCTCCGAAATCTCGTCCATTATCAT<br>TCGCTGGATTTCCGGAGAAAAACAATGACTTCGCTCCGCTACTGGGACATCAG<br>TCCCGCCCTCGACCCGAGCACGCCGACCTGGCCCGGCGACACGCCGTTCCAGC<br>AGGAATG	(1)
gfpmut3	ATGCGTAAAGGAGAAGAACTTTTCACTGGAGTTGTCCCAATTCTTGTTGAATT<br>AGATGGTGATGTTAATGGGCACAAATTTTCTGTCAGTGGAGAGGGTGAAGGTG<br>ATGCAACATACGGAAAACTTACCCTTAAATTTATTTGCACTACTGGAAAACTA<br>CCTGTTCCATGGCCAACACTTGTCACTACTTTCGGTTATGGTGTTCAATGCTT<br>TGCGAGATACCCAGATCATATGAAACGGCATGACTTTTTCAAGAGTGCCATGC<br>CCGAAGGTTATGTACAGGAAAGAACTATATTTTTCAAAGATGACGGGAACTAC<br>AAGACACGTGCTGAAGTCAAGTTTGAAGGTGATACCCTTGTTAATAGAATCGA<br>GTTAAAAGGTATTGATTTTAAAGAAGATGGAAACATTCTTGGACACAAATTGG<br>AATACAACTATAACTCACACAATGTATACATCATGGCAGACAAACAAAAGAAT<br>GGAATCAAAGTTAACTTCAAAATTAGACACAACATTGAAGATGGAAGCGTTCA<br>ACTAGCAGACCATTATCAACAAAATACTCCAATTGGCGATGGCCCTGTCCTTT<br>TACCAGACAACCATTACCTGTCCACACAATCTGCCCTTTCGAAAGATCCCAAC<br>GAAAAGAGAGACCACATGGTCCTTCTTGAGTTTGTAACAGCTGCTGGGATTAC<br>ACATGGCATGGATGAACTATACAAATAA	(2)
DT16 terminator	CTCGGTACCAAATTCCAGAAAAGAGGCCTCCCGAAAGGGGGGCCTTTTTTCGT<br>TTTGGTCCTCCTTGGCCCTCCATCCTTAGATGTCCGGCAATTAAAAAAGCGGC<br>TAACCACGCCGCTTTTTTTACGTCTGCATCATAGGCAATACGATCGCATGTCC	(3)
T7 terminator	CAAAAAACCCCTCAAGACCCGTTTAGAGGCCCCAAGGGGTTATGCTAG	(3)

Biosensor designs and DNA sequences

KYNvA biosensor

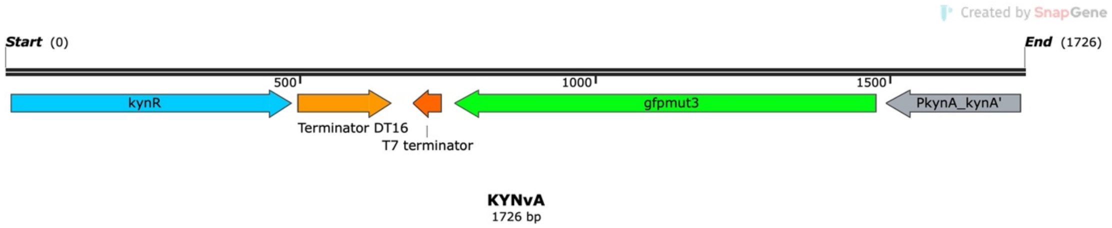

GAATTCACCATGCCCCTGGACGCCATCGATCTGCGCATTCTCCGTCACCTGCAGCAGGACGGGCGGATGAG
CAACCAGGACCTGGCGGAGAGGGTTTCCCTCTCGCCATCCGCCTGCCTGCGCCGGCTGCGCCTGCTGGAAA
GCGAGGGGATCATCCGCGGCTACTCGGTGGAGCTGGATGCCGAGCGTCTGGGCGTGGAGCTGGAAGCCATC
GTCCATGTGTCGCTGCGCCAGGACGTCGAGGGTTGGCACGAGGCGTTCATCGCCAGGGTCCGGGACTGGCC
GGAGGTGGTCACCGCCTATGTGGTGACCGGCGCCACCAACTATGTGCTACGGGTCCGGGCGCGCAACCTCA
AGCACTATTCGGACTTCATCGTGAACAAACTGAACCGCGCCGCCGGCGTCACCGATATCCGCTCGGAAATC
GTCCTGCAGGAGATCAAGGCCGGTGCCGACCTGCTCGACCTGGTGAACCTGAAGAGTTGATAAGAGCTCGC
TCGGTACCAAATTCCAGAAAAGAGGCCTCCCGAAAGGGGGGCCTTTTTTCGTTTTGGTCCTCCTTGGCCCT
CCATCCTTAGATGTCCGGCAATTAAAAAAGCGGCTAACCACGCCGCTTTTTTTACGTCTGCATCATAGGCA
ATACGATCGCATGTCCCCGCCGCCCTAGACCTAGGGTCCGACAAACAACAGCAAAAAACCCCTCAAGACCC
GTTTAGAGGCCCCAAGGGGTTATGCTAGGATCCTTATCTAGAACCTTATTATTTGTATAGTTCATCCATGC
CATGTGTAATCCCAGCAGCTGTTACAAACTCAAGAAGGACCATGTGGTCTCTCTTTTCGTTGGGATCTTTC
GAAAGGGCAGATTGTGTGGACAGGTAATGGTTGTCTGGTAAAAGGACAGGGCCATCGCCAATTGGAGTATT
TTGTTGATAATGGTCTGCTAGTTGAACGCTTCCATCTTCAATGTTGTGTCTAATTTTGAAGTTAACTTTGA
TTCCATTCTTTTGTTTGTCTGCCATGATGTATACATTGTGTGAGTTATAGTTGTATTCCAATTTGTGTCCA
AGAATGTTTCCATCTTCTTTAAAATCAATACCTTTTAACTCGATTCTATTAACAAGGGTATCACCTTCAAA
CTTGACTTCAGCACGTGTCTTGTAGTTCCCGTCATCTTTGAAAAATATAGTTCTTTCCTGTACATAACCTT
CGGGCATGGCACTCTTGAAAAAGTCATGCCGTTTCATATGATCTGGGTATCTCGCAAAGCATTGAACACCA
TAACCGAAAGTAGTGACAAGTGTTGGCCATGGAACAGGTAGTTTTCCAGTAGTGCAAATAAATTTAAGGGT
AAGTTTTCCGTATGTTGCATCACCTTCACCCTCTCCACTGACAGAAAATTTGTGCCCATTAACATCACCAT
CTAATTCAACAAGAATTGGGACAACTCCAGTGAAAAGTTCTTCTCCTTTACGCATGGTGTCGACCTCCTGC
TTAGTGAGGGCAAGGACACATGTGAATCTCCTGAAATTCGGCGGCAGGCGTCGGAAGTCGATGAGCGACCT
GCCAGCAACGTTGTCGGGGGTGGCTTCGACACGGTCGAAGCGCGCTCATTTTACGATGCGCCCATTCGAAT
ATTCGTGCAAAGCATCCTTTCCTCCCCGTCCCCGATGAAATCTAATTCGACGGATCGATGGAAAAATCGAA
TTTCGGATCACTCGCGAAGCTT

KYNvB biosensor

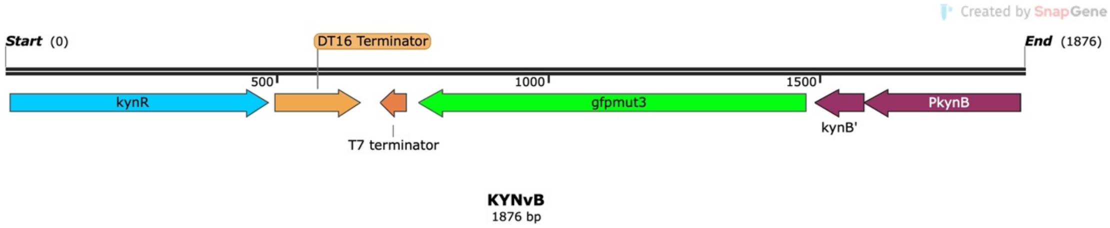

GAATTCACCATGCCCCTGGACGCCATCGATCTGCGCATTCTCCGTCACCTGCAGCAGGACGGGCGGATGAG
CAACCAGGACCTGGCGGAGAGGGTTTCCCTCTCGCCATCCGCCTGCCTGCGCCGGCTGCGCCTGCTGGAAA
GCGAGGGGATCATCCGCGGCTACTCGGTGGAGCTGGATGCCGAGCGTCTGGGCGTGGAGCTGGAAGCCATC
GTCCATGTGTCGCTGCGCCAGGACGTCGAGGGTTGGCACGAGGCGTTCATCGCCAGGGTCCGGGACTGGCC
GGAGGTGGTCACCGCCTATGTGGTGACCGGCGCCACCAACTATGTGCTACGGGTCCGGGCGCGCAACCTCA
AGCACTATTCGGACTTCATCGTGAACAAACTGAACCGCGCCGCCGGCGTCACCGATATCCGCTCGGAAATC
GTCCTGCAGGAGATCAAGGCCGGTGCCGACCTGCTCGACCTGGTGAACCTGAAGAGTTGATAAGAGCTCGC
TCGGTACCAAATTCCAGAAAAGAGGCCTCCCGAAAGGGGGGCCTTTTTTCGTTTTGGTCCTCCTTGGCCCT
CCATCCTTAGATGTCCGGCAATTAAAAAAGCGGCTAACCACGCCGCTTTTTTTACGTCTGCATCATAGGCA
ATACGATCGCATGTCCCCGCCGCCCTAGACCTAGGGTCCGACAAACAACAGCAAAAAACCCCTCAAGACCC
GTTTAGAGGCCCCAAGGGGTTATGCTAGGATCCTTATCTAGAACCTTATTATTTGTATAGTTCATCCATGC
CATGTGTAATCCCAGCAGCTGTTACAAACTCAAGAAGGACCATGTGGTCTCTCTTTTCGTTGGGATCTTTC
GAAAGGGCAGATTGTGTGGACAGGTAATGGTTGTCTGGTAAAAGGACAGGGCCATCGCCAATTGGAGTATT
TTGTTGATAATGGTCTGCTAGTTGAACGCTTCCATCTTCAATGTTGTGTCTAATTTTGAAGTTAACTTTGA
TTCCATTCTTTTGTTTGTCTGCCATGATGTATACATTGTGTGAGTTATAGTTGTATTCCAATTTGTGTCCA
AGAATGTTTCCATCTTCTTTAAAATCAATACCTTTTAACTCGATTCTATTAACAAGGGTATCACCTTCAAA
CTTGACTTCAGCACGTGTCTTGTAGTTCCCGTCATCTTTGAAAAATATAGTTCTTTCCTGTACATAACCTT
CGGGCATGGCACTCTTGAAAAAGTCATGCCGTTTCATATGATCTGGGTATCTCGCAAAGCATTGAACACCA
TAACCGAAAGTAGTGACAAGTGTTGGCCATGGAACAGGTAGTTTTCCAGTAGTGCAAATAAATTTAAGGGT
AAGTTTTCCGTATGTTGCATCACCTTCACCCTCTCCACTGACAGAAAATTTGTGCCCATTAACATCACCAT
CTAATTCAACAAGAATTGGGACAACTCCAGTGAAAAGTTCTTCTCCTTTACGCATGGTGTCGACCTCCTGC
TCATTCCTGCTGGAACGGCGTGTCGCCGGGCCAGGTCGGCGTGCTCGGGTCGAGGGCGGGACTGATGTCCC
AGTAGCGGAGCGAAGTCATTGTTTTTCTCCGGAAATCCAGCGAATGATAATGGACGAGATTTCGGAGAATT
TTCGTGCGAAATGCAAGGCTGCATGGCAATCTGTTCGCACAAAATTCGAAGATAGCTATTCATTCTGATAG
GAATTGCACATGCCCCTGGACGCCATCGATCTGCGCATTCTCCGTCACCTGCAGCAGGACGGGCGGATGAG
CAACCAGGACCTGGCGGAGAGGGTTTCCCTCTCGCCATCCGCCTGCCTGCGCCGGCTGCGCCTGCTGGAAA
GCGAGGGGATCATCCGCGGCTACTAAGCTT

Supplementary References

1. Lee DG, Urbach JM, Wu G, Liberati NT, Feinbaum RL, Miyata S, et al. Genomic analysis reveals that Pseudomonas aeruginosa virulence is combinatorial. Genome Biol. 2006;7(10):R90.
2. Choi KH, Schweizer HP. mini-Tn7 insertion in bacteria with single attTn7 sites: example Pseudomonas aeruginosa. Nat Protoc. 2006;1(1):153-61.
3. Fernandez-Rodriguez J, Moser F, Song M, Voigt CA. Engineering RGB color vision into Escherichia coli. Nat Chem Biol. 2017;13(7):706-8.
